# Novel Recombinant Traceable c-Met Antagonist-Avimer Antibody Mimetic Obtained by Bacterial Expression Analysis

**Published:** 2018

**Authors:** Bahram Baghban Kohnehrouz, Afsaneh Talischian, Alireza Dehnad, Shahnoush Nayeri

**Affiliations:** 1. Department of Plant Breeding & Biotechnology, University of Tabriz, Tabriz, Iran; 2. Department of Biological Sciences, Higher Education Institute of Rab-Rashid, Tabriz, Iran; 3. Department of Biotechnology, East Azerbaijan Research and Education Center Agricultural and Natural Resources, AREEO, Tabriz, Higher Education Institute of Rab-Rashid, Tabriz, Iran; 4. Department of Biotechnology, Shahid Beheshti University, Tehran, Iran

**Keywords:** Antibody avidity, *E. coli* strain BL21, Enzyme-linked immunosorbent assay, Molecular docking analysis

## Abstract

**Background:**

Avimers are originally types of artificial proteins with multiple binding sites for specific binding to certain antigens. Various radioisotopes and nanoparticles link these molecules, which are widely used in early detection in tissue imaging, treatment and study on carcinogenesis. Among these, c-Met antagonist avimer (C426 avimer), with ability to bind the c-Met receptor of tyrosine kinase (RTK) is an attractive candidate for targeted cancer therapy. In this study, a novel traceable C426 avimer gene was designed and introduced by adding the 12nt tracer binding site encoded four specific amino acid residues at the C-terminal region of C426 avimer coding sequence.

**Methods:**

The 282 *bp* DNA sequence encoded 94aa avimer protein was synthesized and sub-cloned into prokaryotic pET26b expression vector. The expression of the mature peptide encoding the traceable avimer molecule was carried out in *Escherichia coli* strain BL21 using IPTG (Isopropyl β-D-1-thiogalactopyranoside) induction process. The expression level of the 11 *kDa* traceable avimer was studied by SDS-PAGE, western blot and ELISA analysis.

**Results:**

Docking analysis of C426 avimer protein and its ligand c-Met showed that the traceability related changes happened at the best conformation and optimal energy. The SDS-PAGE, western blotting and ELISA analysis results demonstrated that the expression of the 11 *kDa* C426 avimer molecule was detectable without any degradation compared with the control group.

**Conclusion:**

Concerning the consequences of this work, this new approach can be widely used in the medical field and provide an opportunity to evaluate the affinity and traceability features.

## Introduction

Recently, a class of antibody mimetics molecules including avimers is developed as the non-immunoglobulin affinity proteins through protein engineering. These molecules are artificial proteins with multiple binding sites that can bind to the specific molecule like antigens. Avimer proteins are composed of two or more peptide sequences with the length of about 30–35aa, which are connected to each other by linker peptides. The main origin of individual peptide sequences is the A domain of various membrane receptors^1^. Domains of this family bind over 100 different known targets, including small molecules, proteins and viruses ^2,3^. The avidity generated by combining multiple binding domains is a powerful approach to increase their affinity and specificity ^4^. These molecules have several advantageous compared to common antibodies including small size, high stability, strong binding affinity to the target molecule, specificity and high resistance against heat denaturation and enzymatic degradation ^5,6^. In addition, the avimer molecules can be produced based on bacterial expression systems ^7^. The development of these kinds of molecules has provided a strong background for production of a variety of avimeric drugs depends on their target cells for clinical uses, treatment of diseases like Crohn’s, autoimmune and various cancers including leukemia and carcinoma.

According to the reports, a number of avimer proteins can bind to the turmeric targets including C426 avimer protein, which has an ability to bind the c-Met receptor. C-Met is a Receptor Tyrosine Kinase (RTK) which is activated upon binding to its only known ligand, Hepatocyte Growth Factor (HGF). Deregulated expression/activation of cMet is found in many neoplasms severity and poor prognosis makes this RTK an attractive candidate for targeted anticancer therapy. Avimer molecules along with various radioisotopes and nanoparticles can be used as biomarkers in tissue imaging too. The imaging of tissue using modern molecular techniques and nuclear medicine has important roles in the early detection of cancer, treatment and study of disease process ^8^.

Hence, the sequence of a novel avimer gene encoding the traceable c-Met antagonist avimer protein was designed and introduced. The expression of the mature peptide of traceable c-Met antagonist avimer molecule was reported in *Escherichia coli (E. coli)* BL21-CodonPlus (DE3)-RIL. It should be noted that this is the first report introducing the traceable c-Met antagonist avimer, which is provided as an opportunity to evaluate the affinity and traceability features of antibody mimetics at future researches of anticancer therapy.

## Materials and Methods

### C426 avimer design

The original amino acid sequence of C426 avimer (PMID: 16299519) was retrieved from GenBank at NCBI (www.ncbi.nlm.nih.gov). The amino acid sequence of C426 avimer was converted into the deduced nucleotide sequence using EMBOSS Backtranseq tool. For prokaryotic expression of the avimer molecule, the appropriate codons were adjusted with codon usage table of *E. coli* and the deduced amino acids sequence was checked out using Expasy translator tool (www.web.expasy.org/translate). Then, the 12nt tracer binding site encoding four specific amino acid residues (glycine, glycine, glycine, cysteine) was added to the 3′end of coding sequence. The cutting restriction sites for nucleotide sequence of C426 avimer were determined using Webcutter (toolrna.lundberg.gu.se/cutter2). The non-cutting restriction sites including *Nco*I/*Nde*I and *Hind*III (arbitrarily defined) were added at the 5′ and 3′ ends of the fragment for sub-cloning into the prokaryotic expression vector, respectively.

### The 3D structure of C426 avimer using homology modelling

The protein secondary structure of designed and original avimer protein was compared by SWISS-MODEL server (http://swissmodel.expasy.org). The three-dimensional structure of the designed avimer protein and its receptor were checked out by ClusPro 2.0 automated web-based program (https://cluspro.bu.edu/home.php). Ramachandran Plot generated by RAMPAGE (http://mordred.bioc.cam.ac.uk) further vali-dated the modelled protein. The physio-chemical properties of the traceable avimer including the molecular weight, theoretical pI, amino acid composition, atomic composition, extinction coefficient, estimated half-life, instability index, aliphatic index, and grand average of hydropathy (GRAVY) were computed by ProtParam tool (http://web.expasy.org/cgi-bin/protparam).

### Identification of C426 avimer protein binding site

Positions of the amino acids present in binding site of the protein and tertiary structure representation of the binding site were determined by FTsite server from Boston University, which is freely available online at http://ftsite.bu.edu/.

### Molecular cloning of C426 avimer

The deduced nucleotide sequence of avimer molecule was synthesized and cloned into pGH cloning vector by GENEray Co. (Shanghai, China). The pET-26b(+) expression vector with kanamycin resistance gene was used for cloning and expression purpose. The DNA fragment of C426 avimer was excised from pGH cloning vector using *Nco*I and *Hind*III restriction enzymes and was recovered by gel purification kit (Bioneer, South Korea; cat. no. k-3035) according to the manufacturer’s instructions. Then, it was sub-cloned into linear pET-26b(+) cloning vector by T4 DNA ligase (Vivantis, USA). The *E*. *coli* strain DH5α competent cells were prepared by TSS reagent and recombinant plasmid was transformed using heat shock method. For screening, the transformed cells were plated on LB-agar medium containing kanamycin antibiotic (50 *μg/ml*). Recombinant plasmid was purified using the plasmid extraction kit (Bioneer, South Korea; cat. no. k-3112). The size of insert was validated by restriction digestion analysis with *Nco*I and *Hind*III and electrophoresis on 1.5% agarose gel. The precision of the ORF region was verified by sequencing analysis (GENEray, China).

### Sequencing and bioinformatics analysis

The sequencing result of pET containing avimer coding sequence was checked out by BLASTn tool at NCBI (https://blast.ncbi.nlm.nih.gov) for the correct positioning of C426 avimer ORF region in the pET-26b (+).

### The expression of the C426 avimer protein

For prokaryotic gene expression study, the pET26-avimer plasmid DNA was transformed into *E. coli* strains BL21 competent cells using heat shock method. The positive colonies were screened out by LB-agar containing kanamycin antibiotic (50 *μg/ml*). Followed by the overnight LB-broth containing kanamycin (50 *μg/ml*) culture of the positive colony, it was treated at 37°*C* for 4 *hr* under 400 *μl* IPTG (10 *mM*) treatment in a shaker incubator at 200 *rpm*. The cells were harvested using 5500 *rpm* centrifuge for 15 *min*. The supernatant obtained after centrifugation and the amount of 2 *ml* of TES buffer was added and mixed well. The cells were harvested three times using sonication method with 25000 *rpm* for 15 *min*.

### SDS-PAGE and western blot analysis

The protein expression analysis was performed by 12% SDS-PAGE and the results were confirmed by a western blot using a His-tag monoclonal antibody as the primary antibody and anti-mouse HRP conjugated immunoglobulin (Abcam, UK) as the secondary antibody.

### Purification of the C426 avimer protein and ELISA procedure

The bacterial lysates were analyzed by 12% sodium dodecyl sulfate-polyacrylamide gel electrophoresis (SDS-PAGE). Based on the His-tag at the N-terminal end, the C426 avimer protein was purified by Ni-NTA affinity chromatography His-Bind Resin according to the manufacturer’s instructions, and stored at −20°*C*. ELISA analysis was conducted to detect expression of protein using anti-His antibody ELISA kit (cat. no. AKR-130; USA). The 96 well micro plates were coated with 100 *μl/well* of the coating solution (Sodium carbonate 50 *mM*, pH=9.6) and were incubated at 4°*C* overnight. The wells were washed five times using washing solution [20 *ml* PBS (1×) +20 *μl* Tween 20], and then were kept at 37°*C* for two *hr*. After adding the 100 *μl* of Anti-His6-Peroxidase solution (10 *mu/ml*) per well, they were incubated at 25°*C* for an *hr*. Wells were washed again and treated with 100 *μl/well* of BM Blue POD Substrate (Roche, Germany), and were incubated at 25°*C* until sufficient color was developed. To stop the color development, 100 *μl/well* of 2N sulfuric acid was added and then their absorbance was determined at 450 *nm*.

## Results

### C426 avimer gene design

**Figure 1.**
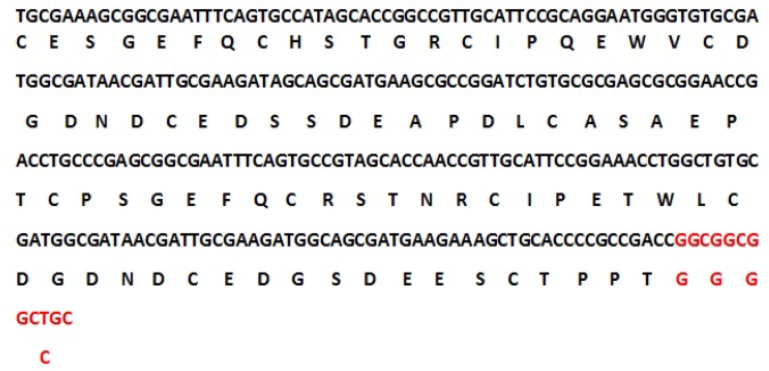
Nucleotide sequence of the traceable C426 avimer gene and its deduced amino acid sequence.

**Figure 2.**
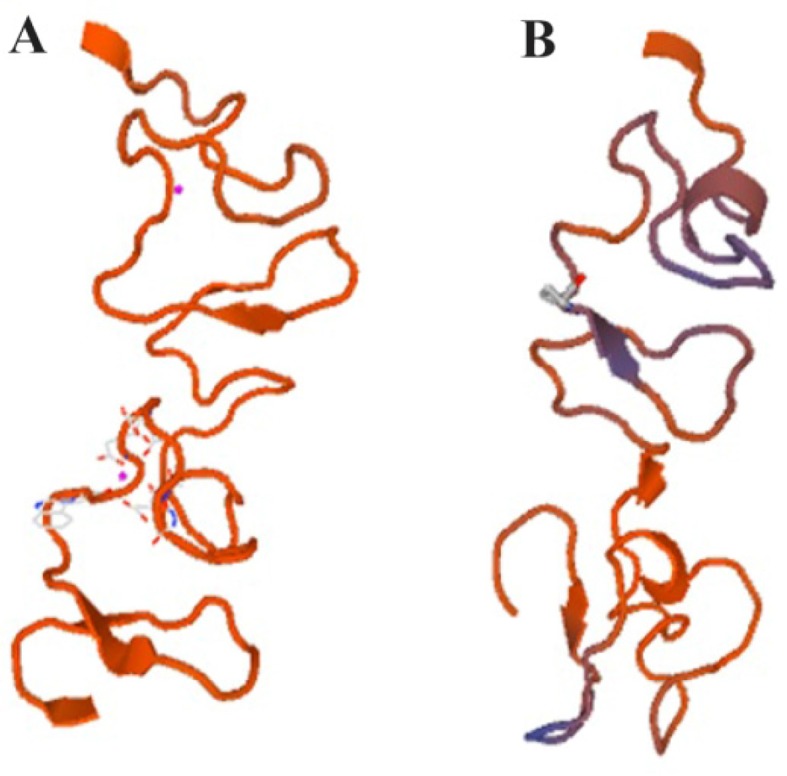
Three-dimensional structure of C426 avimer protein generated by SWISSMODEL. a) Non-traceable avimer protein. b) Traceable avimer protein.

**Figure 3.**
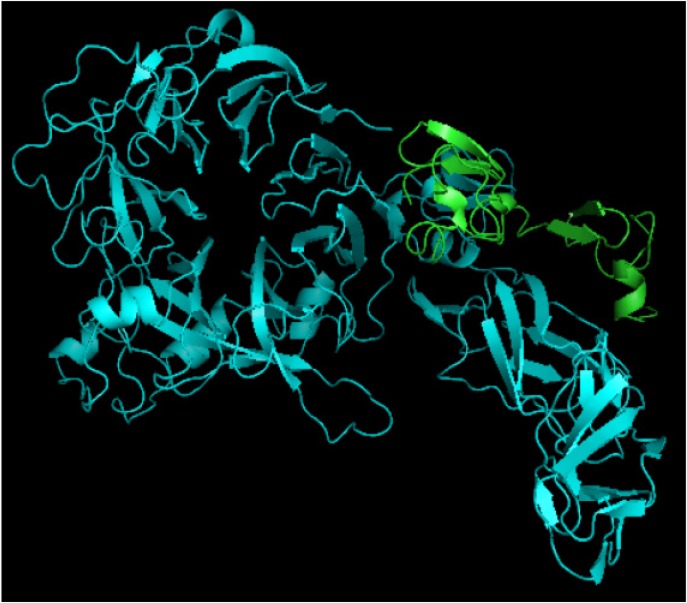
3D structure of avimer-c-Met complex. Blue: c-Met receptor, Green: avimer ligand.

**Figure 4.**
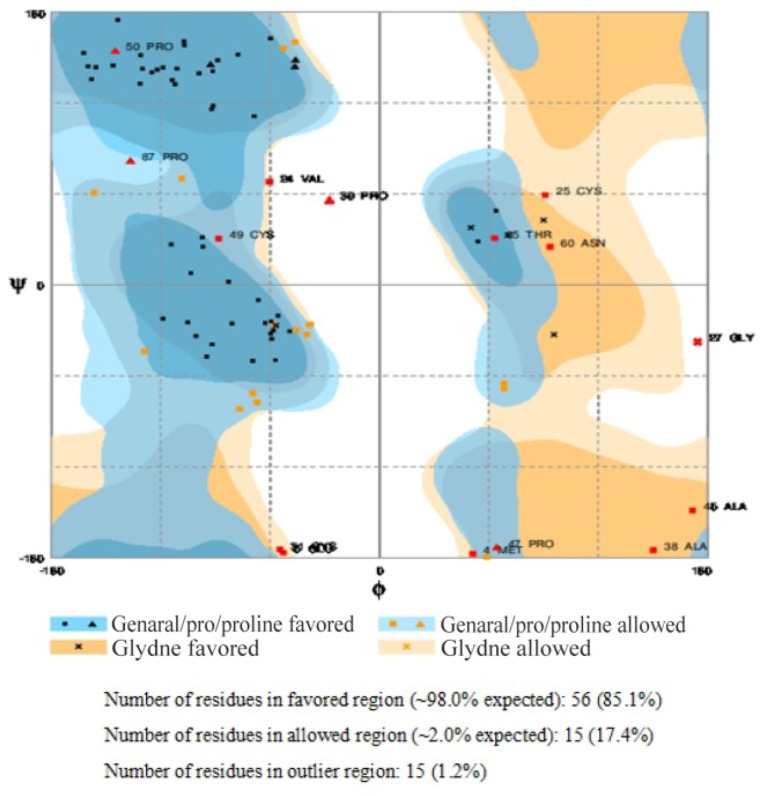
Ramachandran plot generated by RAMPAGE.

The tracer binding DNA linker was added at the 3′-end of the C426 avimer gene (Figure 1). The tertiary structure of the avimer protein was compared with the original avimer protein using SWISS-MODEL server. The tertiary structure of the C426 avimer molecule showed the high similarity of 99% compared to original avimer molecule. The designed tracer binding sites in the sequence of the protein had no effects on the basic structure and molecular functions (Figure 2). In addition, ProtParam was used to analyze different physiochemical properties from the amino acid sequence data of traceable avimer protein. The 94 amino acids long at COP1 protein were predicted to have a molecular weight of 10185.87 *Da* and an isoelectric point (pI) of 3.84. An isoelectric point close to 4 indicates a very negatively charged protein, and an instability index of 47.13 suggests an unstable protein. The negative GRA-VY index of −0.876 is indicative of a hydrophilic and soluble protein. Due to the avimer’s small size, high affinity and stability properties, these molecules can be considered as potential tracers for use in radionuclidebased molecular imaging of tumor-associated antigens. The best variant of tracer binding site -GGGC, provided the lowest radioactivity retention in all normal organs and tissues and the use of a -GGGC sites at the C-terminal end of c-Met antagonist. Avimer enables stable labeling with technetium, with preserved binding specificity. This tracer-binding site can be labeled with radionuclides, fluorescent tag, and Technetium-99m ( ^99m^Tc). The label of ^99m^Tc offers advantages in clinical practice, and earlier studies demonstrated that ^99m^Tc-labeled recombinant antibody mimetics with a C-terminal cysteine could be used for target imaging.^9^ Avimer protein and c-Met interactions were checked out by Cluspro protein-protein docking tool and the server was shown as the best three dimensional structure of avimer-c-Met complex with lowest free ΔG° energy of −972.0. By the Cluspro molecular modeling, the preferred orientation of C426 avimer to c-Met when bound to each other to form a stable complex was predicted and avimer-c-met connection was verified (Figure 3). The modelled protein was further validated by Ramachandran plot generated by RAMPAGE. The plot value was found to be 65.1% with 56 residues in the favored region. 17.4% of the residues lie in additional allowed region 1.6% in generously allowed region. Only about 1.2% of the total residues were located in disallowed region (Figure 4).

### Binding site prediction of C426 avimer

**Figure 5.**
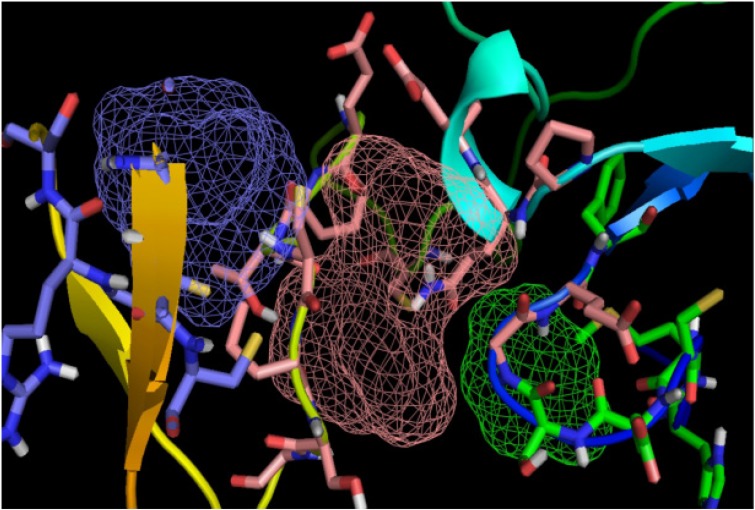
Illustration of the total three numbers of predicted binding sites for C426 avimer protein. Pink, blue and green colored mesh represents the respective first, second and the third binding sites for C426 avimer protein predicted by FTSite server tool.

The potent binding sites of C426 avimer protein was examined by FTsite web server. The results showed that the range of three binding sites constituted amino acid residues, including Gly at 8^th^, Glu at 9^th^, Pro at 20^th^, Gln at 21^th^, Glu at 22^th^, Cys at 42^th^, Glu at 46^th^, Pro at 47^th^, Thr at 48^th^, Cys at 49^th^, Pro at 50^th^, Ser at 51^th^, Gly at 52^th^, positions in the first binding site, His at 3^th^, Met at 4^th^, Cys at 5^th^, Glu at 6^th^, Ser at 7^th^, Gly at 8^th^, Glu at 9^th^, Phe at 10^th^, Gln at 21^th^, positions in the second binding site, Pro at 47^th^, Thr at 48^th^, Cys at 49^th^, Cys at 56^th^, Arg at 57^th^, Ser at 58^th^, Asn at 60^th^, Arg at 61^th^, Cys at 62^th^ positions in the third binding site. The region of binding sites were illustrated using PyMol (Figure 5).

### C426 avimer gene cloning and sequencing

**Figure 6.**
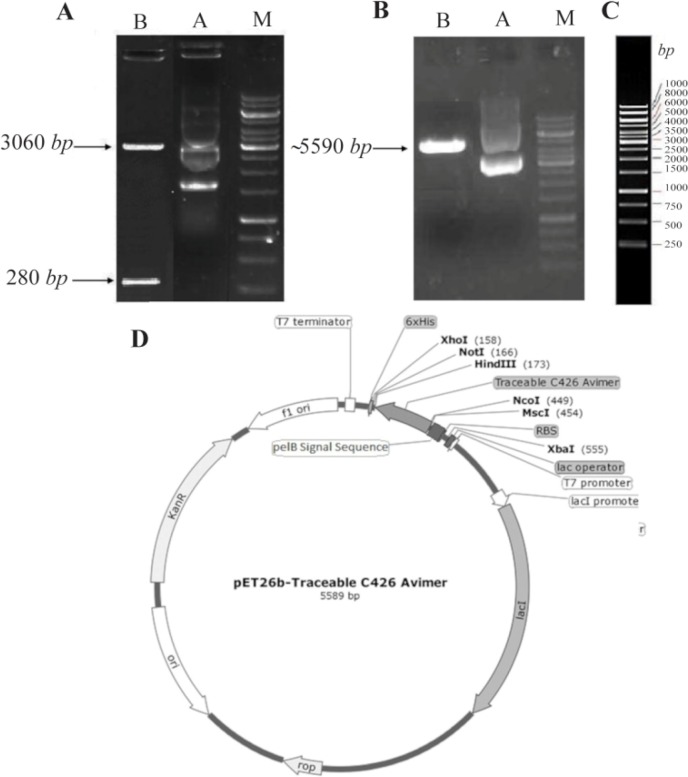
Sub-cloning of C426 avimer gene fragment. A) Screening of plasmid DNA of pET26b-C426 avimer with ∼5590 *bp* length (Lane A) digested by *Hind*III restriction enzyme (Lane B). B) Screening of plasmid DNA of pGH-C426avimer with 360 *bp* length (Lane A) double-digested by *Nco*I/*Hind*III restriction enzymes (Lane B). C) Illustration of gene ruler 1 *kb* DNA ladder (Thermo Scientific Co., USA) with three sharp reference bands (6000, 3000 and 1000 *bp*) loaded on 0.8% agarose gel by Red Safe™ 5% (*v/v*). D) Schematic presentation of the traceable C426 avimer cloned into pET26b+ expression vector with ∼5589 *bp* in length.

The synthesized traceable C426 avimer fragment with ∼280 *bp*-long was excised from plasmid DNA of pGH-C426avimer (∼3340 *bps*) by *Nco*I and *Hind*III double digestion (Figure 6B). Then, it was sub-cloned using a ligation-restriction step into the prokaryotic expression vector pET-26b+(5360 *bps*) by *Nco*I/*Hind*III double digestion (Figure 6D). The avimer fragment size was validated by *Hind*III restriction enzyme using DNA ladder (Lane M) on 0.8% agarose gel electrophoresis (Figure 6A). In figure 5C, the size and concentrations of the reference bands of 1 *kb* DNA ladder are illustrated. T7 promoter primer (TAATACGACTCAC ATTAGGG) presented in figure 2 was used for sequencing. The sequencing result was checked by chromaslite software (http://technelysium.com.au/wp/chromas/). The ORF region of the sequence data was determined using ORF finder software at NCBI. The sequencing result confirmed the sequence data of the designed molecule, comprised of 282 *bp* encoded 94 amino acids of interests.

### SDS-PAGE, western blot and ELISA analysis

**Figure 7.**
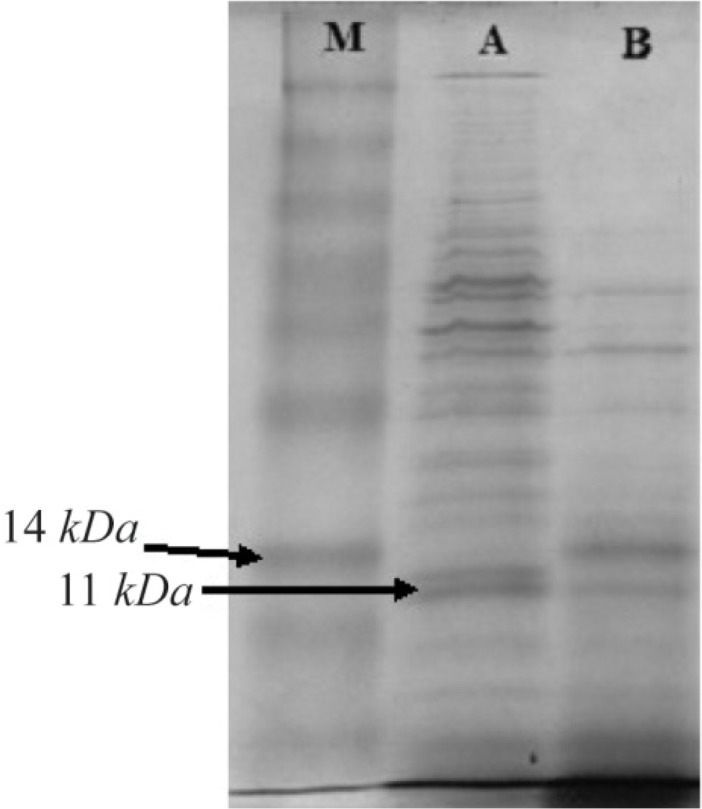
SDS-PAGE analysis of traceable avimer protein. M) Prism ultra-protein ladder, A) Recombinant bacterial protein, B) Non-recombinant bacterial protein complex.

**Figure 8.**
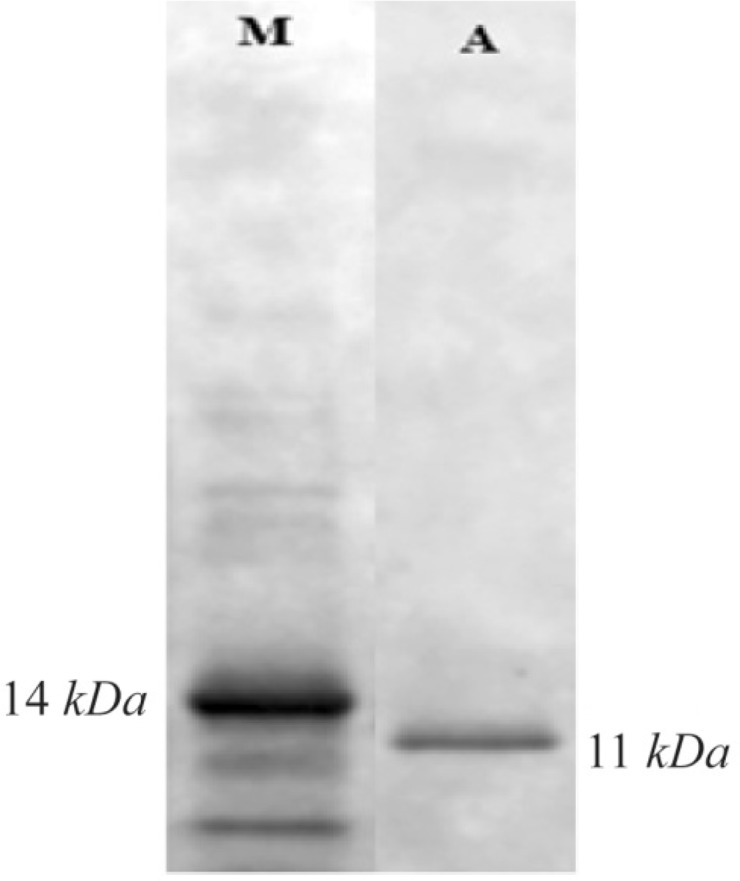
Western blot analysis of expressed protein with His-tag monoclonal antibody.

**Figure 9.**
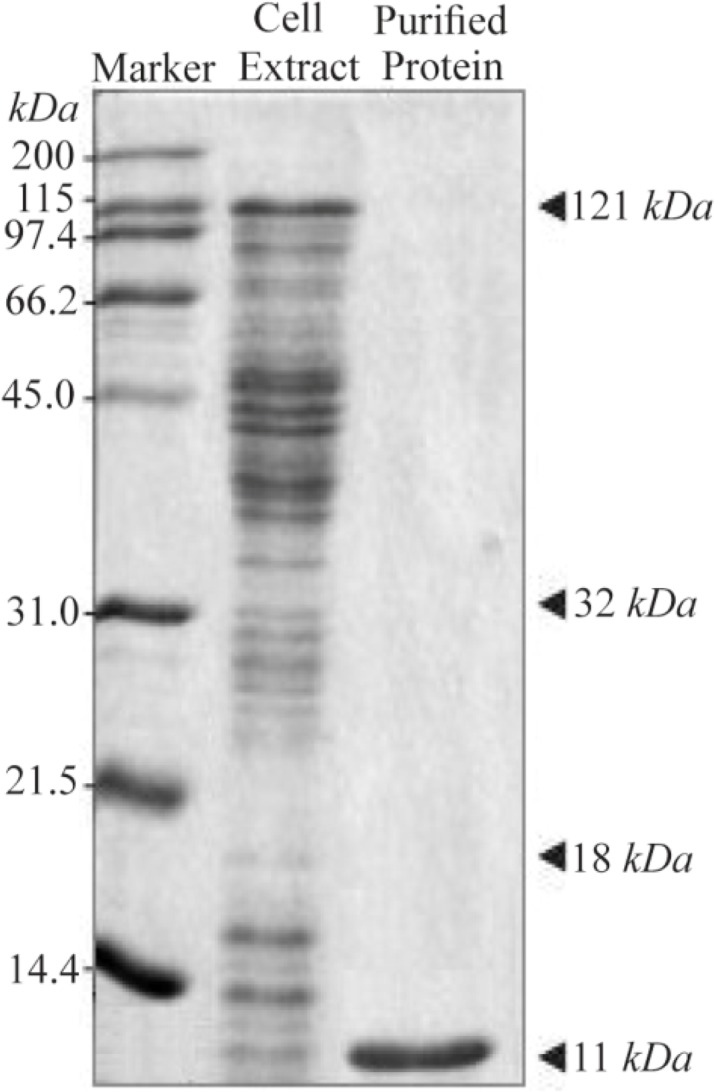
SDS-PAGE gel of bacterial lysate and C426 avimer protein. Size and purity of the recombinant protein were evaluated using 12% SDS-PAGE gel. The avimer protein for ELISA analysis was purified using anti-His antibody ELISA kit.

The C426 avimer recombinant protein was expressed under IPTG induction and detected using SDS-PAGE (according to band abundance the concentration of protein is predicted to be 5 *μg/μl*) and western blotting. The expression of the 11 *kDa* expected protein was detected without any degradation compared to the control groups in SDS-PAGE method and western blotting analysis, respectively as the non-recombinant bacterial protein extraction (Figure 7, Lane B) and the ultra-protein ladder (Figure 8), which are showing lack of any signal. The ELISA analysis result showed the higher value of 2.125±0.231 in comparison to the results of bacterial lysate with value of 0.085±0.015 (p< 0.001) which indicates the presence of recombinant protein (Figure 9).

## Discussion

Nowadays, there is clear evidence that most of therapeutic antibodies have some usage limitations including low half-life, large size, and weak stability against thermal and enzymatic degradations and inappropriate reactions against the target molecules. Antibody mimetics are organic compounds like antibodies, and they can specifically bind antigens, but are not structurally related to antibodies. They are usually artificial peptides or proteins with a mass of 3 to 20 *kDa*. Common advantages of these molecules over antibodies are better solubility, tissue penetration, stability against heat and enzymes, and comparatively low production costs. Recently, antibody mimetics are being developed as therapeutic and diagnostic agents ^10^. One of these antibody mimetics is called avimer. These are evolved from a large family of human extracellular receptor domains. The ability to inhibit more than one target using a single molecule can be a major advantage in therapeutic applications. The modular nature and consistent physical properties of avimers simplifies incorporation of multiple specificities into a single molecule ^11^. Avimers are highly resistant to temperature denaturation and the half-life of this in human is in the range of several approved antibody drugs ^12^. Traceability is a major factor in following any biotherapeutics in the body. Thus, many researchers have conducted extensive researches on the antibody mimetics and their affinity to counterpart molecules. In the present study, an attempt was made to express recombinant avimer. A class of binding proteins, called traceable c-Met antagonist avimers, was developed to overcome the limitations of general antibodies and other immunoglobulinbased therapeutic proteins. However, Silverman *et al* used the phage display technique for this goal. Notably, antibody inhibitors to cMet have been historically difficult to obtain, because the divalent nature of a whole antibody leads to receptor dimerization and undesirable agonistic effects. In this study, an 11 *kDa* band was observed by SDS-PAGE as well as western blotting. Based on the results of this study, it was concluded that the C426 avimer gene could be cloned and expressed using the bacterial protein expression system which can be used for the early detection and treatment of cancer and the study of disease process as well.

## Conclusion

This experiment provided evidence that traceable C426 avimer gene coding this protein can be expressed in *E. coli*. In the future, this protein should be used (radionuclide) for molecular imaging of tumor-associated antigens, early detection of cancer, treatment and study of disease processes.
